# Binding Studies of Caffeic and p-Coumaric Acid with α-Amylase: Multispectroscopic and Computational Approaches Deciphering the Effect on Advanced Glycation End Products (AGEs)

**DOI:** 10.3390/molecules27133992

**Published:** 2022-06-21

**Authors:** Mohd Shahnawaz Khan, Majed S. Alokail, Amal Majed H. Alenad, Nojood Altwaijry, Nouf Omar Alafaleq, Abdulaziz Mohammed Alamri, Mubarak Ali Zawba

**Affiliations:** Department of Biochemistry, College of Science, King Saud University, Riyadh 11451, Saudi Arabia; malokail@ksu.edu.sa (M.S.A.); aalenad@ksu.edu.sa (A.M.H.A.); nojood@ksu.edu.sa (N.A.); nalafaleg@ksu.edu.sa (N.O.A.); abalamri@ksu.edu.sa (A.M.A.); 436160113@student.ksu.edu.sa (M.A.Z.)

**Keywords:** α-amylase, advanced glycation end-products, caffeic acid, coumaric acid

## Abstract

Alpha-amylase (α-amylase) is a key player in the management of diabetes and its related complications. This study was intended to have an insight into the binding of caffeic acid and coumaric acid with α-amylase and analyze the effect of these compounds on the formation of advanced glycation end-products (AGEs). Fluorescence quenching studies suggested that both the compounds showed an appreciable binding affinity towards α-amylase. The evaluation of thermodynamic parameters (Δ*H* and Δ*S*) suggested that the α-amylase-caffeic/coumaric acid complex formation is driven by van der Waals force and hydrogen bonding, and thus complexation process is seemingly specific. Moreover, glycation and oxidation studies were also performed to explore the multitarget to manage diabetes complications. Caffeic and coumaric acid both inhibited fructosamine content and AGE fluorescence, suggesting their role in the inhibition of early and advanced glycation end-products (AGEs). However, the glycation inhibitory potential of caffeic acid was more in comparison to p-coumaric acid. This high antiglycative potential can be attributed to its additional –OH group and high antioxidant activity. There was a significant recovery of 84.5% in free thiol groups in the presence of caffeic acid, while coumaric attenuated the slow recovery of 29.4% of thiol groups. In vitro studies were further entrenched by in silico studies. Molecular docking studies revealed that caffeic acid formed six hydrogen bonds (Trp 59, Gln 63, Arg 195, Arg 195, Asp 197 and Asp 197) while coumaric acid formed four H-bonds with Trp 59, Gln 63, Arg 195 and Asp 300. Our studies highlighted the role of hydrogen bonding, and the ligands such as caffeic or coumaric acid could be exploited to design antidiabetic drugs.

## 1. Introduction

In the past decades, rigorous research and management strategies have been implicated in tackling the menace of diabetes mellitus (DM) [1]. According to the International Diabetes Federation, the number of diabetic patients reached 463 million in 2019, and the numbers are expected to rise by 66% by 2045 [2]. DM is a metabolic syndrome characterized by a loss of capability to oxidize carbohydrates by individuals due to altered insulin production, low insulin production and desensitized insulin receptors [3]. The inefficiency in metabolizing carbohydrates causes a surge in glucose levels in the patients’ blood. Monomeric carbohydrates can undergo non-enzymatic glycation towards proteins, nucleic acids and lipids [4].

Glycation is a non-enzymatic and spontaneous reaction. It is commenced by the reaction between reducing sugars carbonyl group with a free amino group, generally lysine’s ɛ-amino group and α-amino group of the protein. The process forms complex adducts referred to as Schiff base [5]. The base undergoes Amadori rearrangement to form various complex compounds known as advanced glycation end products (AGEs) [6,7]. There are a series of events that results in cellular malfunctioning as a result of high glucose, and these are not fully elucidated, thereby attracting researchers across the globe. Amongst these, a major event is the formation of AGEs that can contribute to diabetic complications in different ways. Glycation of proteins results in AGEs formation, leading to many diabetic complications. Protein glycation alters their normal functioning in various ways, viz. disruption of molecular conformation, alteration of enzymatic activity, reducing degradation capacity and others. AGEs interaction with their cellular receptors plays a crucial role in the pathogenesis of diabetic complications [8]. AGEs formation further aids the formation of reactive oxygen species (ROS), which further damages the cellular antioxidant defense mechanism and increases the oxidative load [9]. AGE formation is augmented in diabetic conditions, especially in Type 2 DM [10,11]. Glycation contributes greatly to AGE formation; highly reactive C=O species such as 3-deoxyglucosone and methylglyoxal are generated in glycation, lipid peroxidation and polyol pathway of DM type II [12]. Accumulation of AGEs contributes to many chronic diseases such as diabetes, Alzheimer’s disease, heart failure and many other life-threatening conditions [13]. Further AGEs interaction with RAGEs, their specific receptors causes an onset of an inflammation cascade and further increases the oxidative load in the cells. The cascade of inflammation involves the activation of several pathways involved in diabetes, such as the MAP kinase pathway, TNF-α, NF-Kb, etc. [14]. The toxic accumulations can further contribute to pathogenesis in diabetic conditions such as decreased ligand binding, altered enzymatic activities and immunogenicity [15].

α-Amylase is an essential enzyme in carbohydrate degradation and is linked with postprandial hyperglycemia in Type-II DM patients. The enzyme hydrolyzes carbohydrates and creates a glycemic environment in the bloodstream, aiding AGEs formation and thrusting diabetic complications [16]. Constraining these enzymes’ activity can subdue blood glucose levels. Investigations have shown that α-amylase inhibition aids in regulating glucose levels [17]. Polyphenols, their derivatives and other naturally derived compounds show therapeutic potential against various diseases such as cancer [18,19,20,21], neurodegenerative disorders [22,23], diabetes [24,25] and many more.

Caffeic acid (3,4-dihydroxycinnamic) is one of the most abundant hydroxycinnamate present in plant tissues. Phenolic acid caffeine is prevalent in various food sources such as apple, cider, blueberries, etc., and beverages such as tea and coffee [26]. Caffeic acid is known to cross the brain barrier and is classified as an antioxidant, antibacterial and anti-cancer compound [27,28]. Similarly, Coumaric acid is an essential polyphenol that governs the synthesis of some other important polyphenolic compounds such as sinapinic, ferulic and caffeic acid [29,30]. CA also plays an important role in lignin synthesis and is an abundant dietary polyphenol present in apples, maize, tomato and wheat [31]. The present study was designed to explore the binding mechanism of polyphenols (caffeic/coumaric acid) to α-amylase. Our study was also extended to reveal the anti-glycation activities of these structures and their modes of action. Various spectroscopic, biochemicals and computational approaches were employed.

## 2. Materials and Methods

### 2.1. Materials

Human serum albumin (HSA), methylglyoxal (MG), nitro blue tetrazolium (NBT), 2,4-Dinitrophenylhydrazine (DNPH), caffeic acid, p-coumaric acid and porcine pancreatic α-amylase were purchased from Sigma-Aldrich, Milwaukee, WI, USA. All other chemicals, unless stated otherwise, were high grade and purchased from local vendors. All the blanks were run replicating the same conditions and acted as a control. All the buffers were filtered before use.

### 2.2. Steady-State Fluorescence

The inhibitory effect of caffeic and coumaric acid on α-amylase was studied by exploiting the fluorescence ability of the protein. Quenching studies were carried out against the protein in the presence of both the natural compounds, using a spectrofluorometer (Jasco FP-750, JASCO Corporation, Tokyo, Japan). The protein (5 μM α-amylase) was excited at 295 nm, and the emission spectra were recorded at higher wavelengths between 300–400nm [32,33,34,35]. Caffeic acid (0–40 μM) and coumaric acid (0–30 μM) were titrated with the α-amylase at three different temperatures (25, 30 and 35 °C). All these binding experiments were conducted at physiological pH of 7.4. The obtained quenching data were put into Stern–Volmer (Equation (1)) and modified Stern–Volmer equation (Equation (2)) as per earlier published literature [20,21,36] to find various binding parameters. The binding experiment was performed at physiological pH of 7.4.

*F*_0_ corresponds to the maximum fluorescence intensity of free protein, *F shows the fluorescence intensity of complex, *K corresponds to the binding site, *n* depicts the binding sites and *C refers to the concentration of ligand.

*F_0_/F* = 1 + *K*sv [*C*] (1)

*log [(F*_0_ *− F)/F] = log K + nlog*[*C*] (2)

Using the van’t Hoff equation, (Equation (3)) [37,38], thermodynamic parameters for ligand–protein interaction such as Gibbs free energy change (Δ*G***⁰**), enthalpy change (Δ*H***⁰**) and entropy change (Δ*S***⁰**) can be calculated.

Δ*G***⁰** = −RTLn*K* = Δ*H***⁰** − TΔ*S***⁰** (3)

The mode of quenching was further confirmed from the value of the bimolecular quenching rate constant, *K*_q_, which was calculated as per Equation (4)

*K*q = *K*sv/τ_o_ (4)

τ_o_ refers to the average integral fluorescence lifetime of tryptophan and is reported to be 10^−8^.

### 2.3. Synchronous Fluorescence

Synchronous fluorescence spectrometry is used as a common tool to inspect conformational changes in proteins in the presence of other molecules, also termed quenchers [39]. Synchronous fluorescence analysis of α-amylase was carried out in the absence and presence of caffeic acid and coumaric acid (0–20 μM) to obtain the spectra. A 15 nm difference was kept for Tyr residues between the excitation (245 nm) and emission spectra (260–340 nm). In a similar manner for Trp, the difference was maintained at 60 nm, the excitation was fixed at 220 nm and the emission was between 280–400 nm.

### 2.4. Non-Enzymatic Glycation

HSA was glycated using methylglyoxal (MG) as an inducer, as reported earlier [36,40]. Briefly, HSA was taken at the concentration of 10 mg/mL and incubated along with MG (3 mM) in the presence of caffeic and coumaric acid (0–200 µM) under sterile conditions using 0.02% sodium azide. HSA alone and in the presence of MG was also incubated under similar conditions as negative and positive control samples, respectively. Samples were further dialyzed in 20 mM sodium phosphate buffer with successive changes at regular intervals for 24 h. Protein concentration was determined using the Bradford method [41] and stored at −20 °C.

### 2.5. Determination of Advanced Glycation End-Products (AGEs)

AGEs were estimated for all the samples using fluorescence spectroscopy. A dilution factor of 10 was applied to all the samples, and then, the samples incubated were excited at 340 nm, and the emission was recorded from 350 to 500 nm [40]. The inhibitory effect of the ligand on the AGEs formation was calculated by the given Equation (5):

*Inhibition* (%) = (*F_g_* − *F_t_*/*F_g_* − *F_c_*) × 100 (5)

### 2.6. Detection of Early Glycation (Amadori) Products: Quantification of Fructosamine

NBT assay was used to determine fructosamine content; the previously used protocol was used [42]. Briefly, 0.5 mM NBT was mixed with samples (0.5 mg/mL) and incubated in 100 mM sodium carbonate buffer of pH 10.4. The reaction mixture was incubated for 2 h at 37 °C, and reading was taken at 530 nm. The concentration of fructosamine was evaluated using its molar extinction coefficient value, i.e., 12,640 M^−1^ cm^−1^ [43].

### 2.7. Protein Oxidation Measurement: Carbonyl and Free Thiol (SH) Content

Carbonyl content was estimated to calculate the level of protein oxidation [42]. Briefly, aliquoted protein samples (100 μL) were mixed with 400 μL DNPH (10 mM). After thorough mixing, 500 μL of TCA (20% *w/v*) was added and centrifuged at 10,000× *g* for 10 min. The pellet was washed further with a 1 mL ethanol/ethyl acetate (1:1) mixture and resuspended in 1 mL of 6 M guanidine hydrochloride. The absorbance of the sample was recorded at 370 nm, and the concentration expressed as nanomoles of carbonyls per milligram of protein was determined using 22,000 M^−1^ cm^−1^ as molar absorptivity.

Ellman’s reagent was used to calculate the free thiol content [44]. Native and glycated samples in the absence and presence of caffeic/coumaric acid (250 μL) were incubated with 750 μL of DTNB (0·5 mM) for 15 min, and the absorbance was measured at 412 nm. The concentration of free thiol groups was calculated using a standard curve of L-cysteine and expressed as nanomoles of L-cysteine per milligram of protein.

### 2.8. Molecular Docking

The interaction between pancreatic α-amylase and both caffeic acid and p-coumaric acid was performed using Autodock-4.2.6 and Discovery. The three-dimensional coordinates of pancreatic α-amylase (PDB ID: 1hny) were retrieved from the protein data bank (www.rcsb.org, accessed on 22 February 2022). The X-ray structure was 1.8 Å [45]. The enzyme structure was pre-processed by adding polar hydrogen atoms, deleting unessential water molecules, and adding Kollman charges through Autodock. Similarly, the two-dimensional structures of both caffeic acid and p-coumaric acid were downloaded from the PDB website (CID: 689043 and CID: 637542, respectively). The ligands were prepared through Autodock, and Gasteiger charges were added. Molecular docking was performed through Autodock using a genetic algorithm, and the output was set to Lamarckian GA (4.2).

## 3. Results and Discussion

### 3.1. Intrinsic Fluorescence

**Figure 1:**
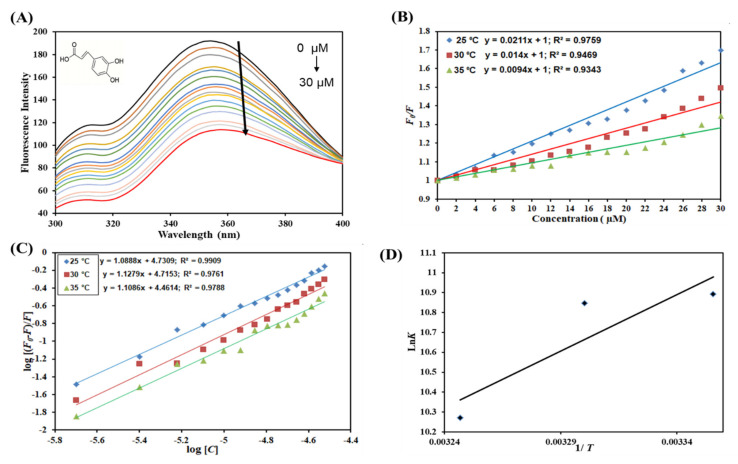
Binding between caffeic acid and α-amylase. (**A**) Quenching in fluorescence intensity of α-amylase (5 µM) in the absence and presence of varying caffeic acid concentration (0–30 µM) at 298 K, (**B**) Stern–Volmer plot at different temperatures, (**C**) modified Stern–Volmer plot at different temperatures, and (**D**) van’t Hoff thermodynamics plot at three different temperatures.

**Figure 2:**
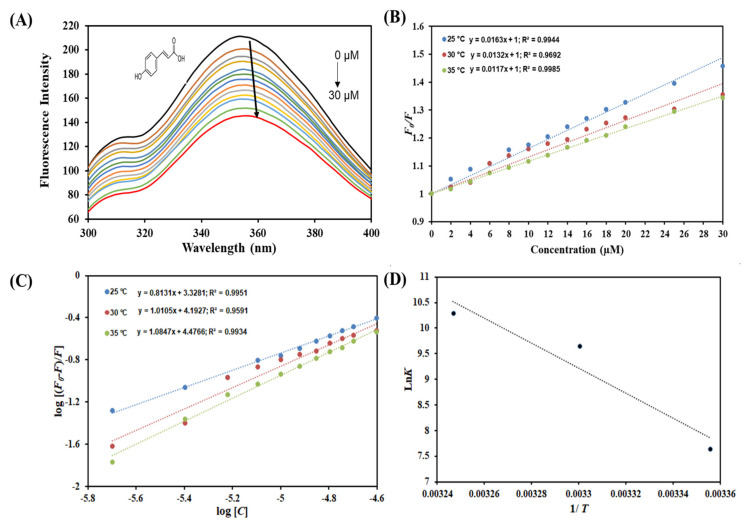
(**A**) Binding between p-coumaric acid and α-amylase. Fluorescence quenching intensity of α-amylase (5 µM) in the absence and presence of varying p-coumaric acid concentration (0–30 µM) at 298 K, (**B**) Stern–Volmer plot at different temperatures, (**C**) modified Stern–Volmer plot at different temperatures and (**D**) van’t Hoff thermodynamics plot at three different temperatures.

The pattern of changes exhibited in the protein fluorescence after adding a ligand provides information with structure and hence the protein’s function [46]. Steady-state fluorescence quenching has been used to determine various binding parameters of ligand–protein interactions. Trp residue has the strongest fluorescence intensity and is the most sensitive to the changes in the micro-environment [47], and its emission wavelength is more sensitive to the microenvironment, indicating the protein conformational changes after binding with drugs [48]. Generally, the quenching mechanism can be classified into three categories: (1) dynamic quenching, which is caused by the collision between molecules in the transition to the excited state; (2) static quenching, which is caused by the formation of a complex between the fluorophore and the quencher; (3) the combined static and dynamic quenching [49]. The quenching was carried out with increasing concentrations of the ligands against α-amylase at different temperatures (Figure 1A and Figure 2A). As indicated by the spectra, quenching was observed with increasing concentration of both the ligands caffeic and coumaric acid. This decrease in the fluorescence intensity amid the addition of caffeic acid and coumaric acid indicates that the ligands interact in the microenvironment of aromatic residues of α-amylase and mask the internal optimal fluorescence intensity. Previous investigations have shown that many other polyphenols exhibited similar behavior, thereby decreasing the fluorescence intensity of α-amylase [29,37,38]. Fluorescence quenching data were evaluated mathematically employing Stern–Volmer, modified Stern–Volmer, and van’t Hoff equations to calculate the binding and thermodynamic parameters [26,28].

In the SV plot of *F*_0_*/F* vs. caffeic acid and *F*_0_*/F* vs. [coumaric acid] (Figure 1B and Figure 2B), the slope gives the value of the Stern–Volmer constant (*K*_sv_) [40]. Figure 1B and Figure 2B apparently show linear SV plots for caffeic acid–α-amylase and coumaric acid–α-amylase, respectively. Fluorescence quenching can be static or dynamic or a combination of both [34,35]. Temperature dependency of *K*_sv_ reveals the type of quenching operative for a particular interaction, i.e., protein–ligand complex formation is driven by static or dynamic quenching. The *K*_sv_ value decreases with increasing temperature for static quenching due to complex formation, which undergoes dissociation on increasing the temperature. In contrast, the opposite effect occurs for dynamic quenching, where *K*_sv_ increases with temperature. Hence, the values of *K*_sv_ were estimated at three different temperatures for α-amylase-caffeic acid and α-amylase-coumaric acid and are enumerated in Table 1 and Table 2, respectively. *K*_sv_ increases with increasing temperature for α-amylase–caffeic acid interaction, implying dynamic quenching. On the contrary, *K*_sv_ values were found to decrease with increasing temperature for α-amylase–coumaric acid, suggesting the presence of static quenching. These observations can corroborate previously reported results [30,40]. Further, the quenching mode was confirmed by finding the biomolecular quenching rate constant (*K*_q_) using the equation *K*_q_ = *K*_sv_/τ_0_ (τ_0_ = 2.7 × 10^−9^ s). The values of *K*_q_ for α-amylase–caffeic acid (Table 1) and α-amylase–coumaric acid (Table 2) were found to be higher than the maximum dynamic quenching constant (nearly 10^10^ M^−1^ s^−1^) [49]. Thus, it can be concluded that α-amylase–caffeic acid complex formation was driven by dynamic quenching while a combination of static and dynamic directs α-amylase–coumaric acid complex formation, while α-amylase–coumaric acid interaction was driven by static quenching. Additionally, the binding constant (*K*) was also calculated, revealing the binding affinity for protein (Table 2). Fluorescence quenching data were fitted into a modified Stern–Volmer equation with the intercept of the plot giving the value of binding constant (*K*) for both the ligands (Figure 1C and Figure 2C). It was found to be of the order of 10^4^ M^−1^ for α-amylase–caffeic acid complex, while for α-amylase–coumaric acid, *K* was of the order of 10^4^ M^−1^, but lesser than caffeic acid, suggesting that caffeic acid binds to α-amylase with a higher affinity as compared to coumaric acid. Table 1 depicts the values of *K* at different temperatures for α-amylase–caffeic acid complex, which was found to decrease with increasing temperature, implying that a more stable complex is formed at lower temperatures. Table 2 depicts the values of K obtained for α-amylase–coumaric acid complex.

The data obtained at different temperatures are fitted into the van’t Hoff equation to find the thermodynamic parameters of the system for both the ligands (Figure 1D and Figure 2D). The slope of this plot gives −Δ*H*/R, and the intercept gives the value of Δ*S*/R. The magnitude and the sign of the thermodynamic parameters (Δ*H*, Δ*S* and Δ*G*) offer a clue about the forces that drive the reaction. Table 1 shows the various thermodynamic parameters obtained for the α-amylase–caffeic acid system, while Table 2 shows the thermodynamic parameters obtained for the α-amylase–coumaric acid system. We obtained negative Δ*H* and Δ*S* for the α-amylase–caffeic acid system, revealing the existence of van der Waals force and hydrogen bonding, while positive Δ*H* and Δ*S* were obtained for α-amylase–coumaric acid, implying the reaction to be driven predominantly by hydrophobic interaction [50,51]. Additionally, negative ΔG for both the systems suggested the spontaneous nature of the reaction.

### 3.2. Synchronous Fluorescence

**Figure 3:**
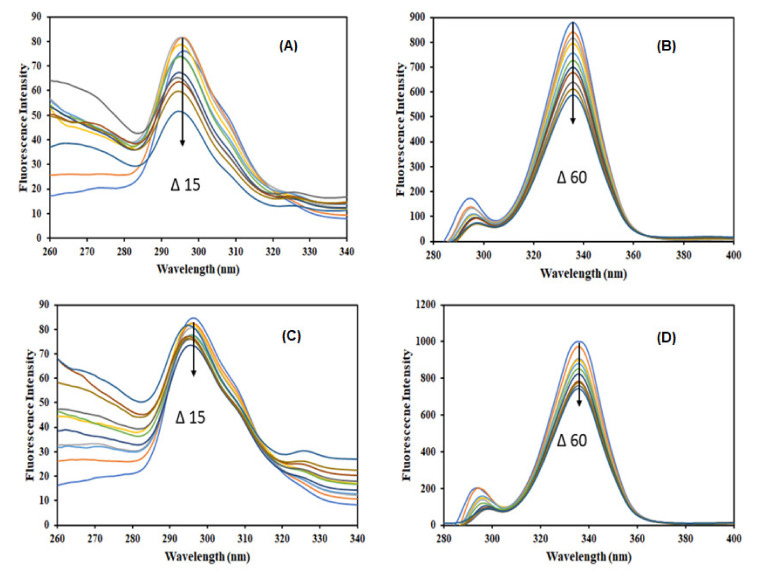
Synchronous fluorescence spectra for (**A**) Tyrosine residue (Δλ = 15) and (**B**) Tryptophan (Δλ = 60) of α-amylase (4 µM) in the absence and presence of caffeic acid (0–40 µM). Panel (**C**,**D**) are the spectra obtained under similar conditions for p-coumaric acid.

Synchronous fluorescence spectroscopy is used to have deeper insights into the conformational changes in the proteins microenvironment comprising tyrosine and tryptophan residues. Synchronous fluorescence provides information on conformational changes in the molecular environment of fluorophores of proteins once the ligands bind to them [52]. When Δλ (λ_em_ − λ_ex_) is kept at 60 nm or 15 nm, the synchronous fluorescence spectra expose the information about the microenvironment of tryptophan and tyrosine residues, respectively. Figure 3 shows the synchronous fluorescence spectra of free α-amylase and α-amylase with varying concentrations of caffeic acid (upper panel) and coumaric acid (lower panel).

In the case of Δλ = 15 nm, a shift in the fluorescence emission maxima of α-amylase in the presence of caffeic acid (Figure 3A) and coumaric acid (Figure 3C) implies that the local environment around tyrosine residue changed significantly in the presence of both the ligands. However, for Δλ = 60 nm, no shift in the emission maxima of α-amylase is observed for both the ligands (Figure 3B,D), suggesting no change in the local environment around tryptophan residues.

### 3.3. Inhibition of Advanced Glycation End-Products (AGEs)

**Figure 4:**
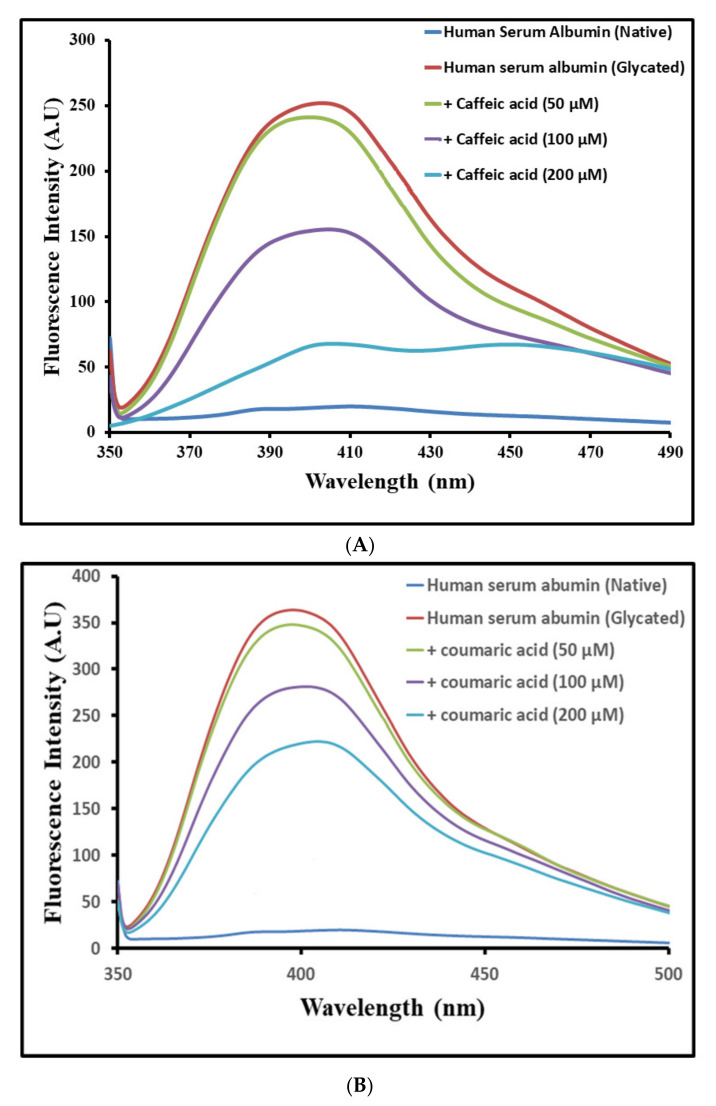
Inhibition of fluorescent advance glycation product (AGEs) by (**A**) caffeic acid and (**B**) p-coumaric acid.

AGE formation is common at the later glycation stages in proteins, and most of the end products are fluorogenic in nature [53]. Caffeic acid and coumaric acid were studied for their inhibitory effects on AGE formation (Figure 4). HSA was incubated with MG, fluorescence was taken of native HSA, and MG + HSA was incubated. HSA incubated with MG showed high fluorescence compared to the native HSA, concluding the formation of AGEs. In the presence of varying concentrations (50–200 μM) of ligands, a dose-dependent decrease in the fluorescence emission was evident, with maximum inhibition observed for 200 μM for both the ligands (Figure 4A).

Treatment of MG incubated HSA in the presence of 50, 100 and 200 μM ligands showed a reduction in fluorescent AGEs. Caffeic and coumaric acid both showed a significant decrease (79.2% and 43.6%) respectively at 200 µM concentration (Figure 4A,B). The antiglycative properties of caffeic acid and coumaric acid have been investigated in previous studies [53,54]. AGEs are the key players in the pathophysiology and progression of many diseases highlighting their clinical significance [30]. Strong inhibition of AGEs by caffeic acid compared to coumaric acid could be due to structural differences among them, where caffeic acid possesses 1 more OH group and might play an important role in AGE inhibition.

The results indicated that the binding of caffeic acid and coumaric acid inhibits the formation of AGEs. Thus, a conclusion was obtained that both the ligands attenuate the effect of MG by networking with HSA and reduce the fluorescence by AGEs. AGEs not only create a menace in diabetes but also contribute to other fatal diseases [29,53], signifying an urgent need to stop AGEs formation [54]. Antiglycation activities of caffeic and coumaric acid could be due to its antioxidant, ROS scavenging activity and protein-stabilizing potential. Earlier, caffeic and ferulic acid have been found to attenuate glycation and thus diabetic complications [55,56]. Additionally, the binding analysis of these phenolics with α-amylase hypothesized its inhibitory potential and thereby reduced glucose concentrations in serum.

### 3.4. Inhibition of Early (Amadori) Glycation Products

**Figure 5:**
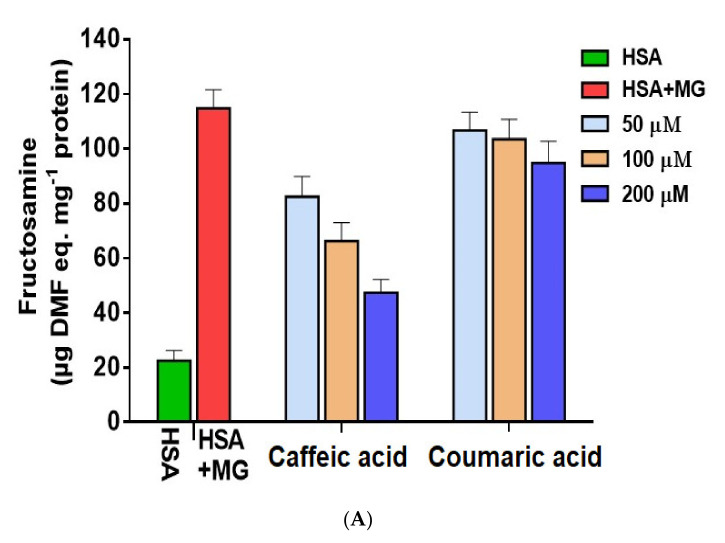

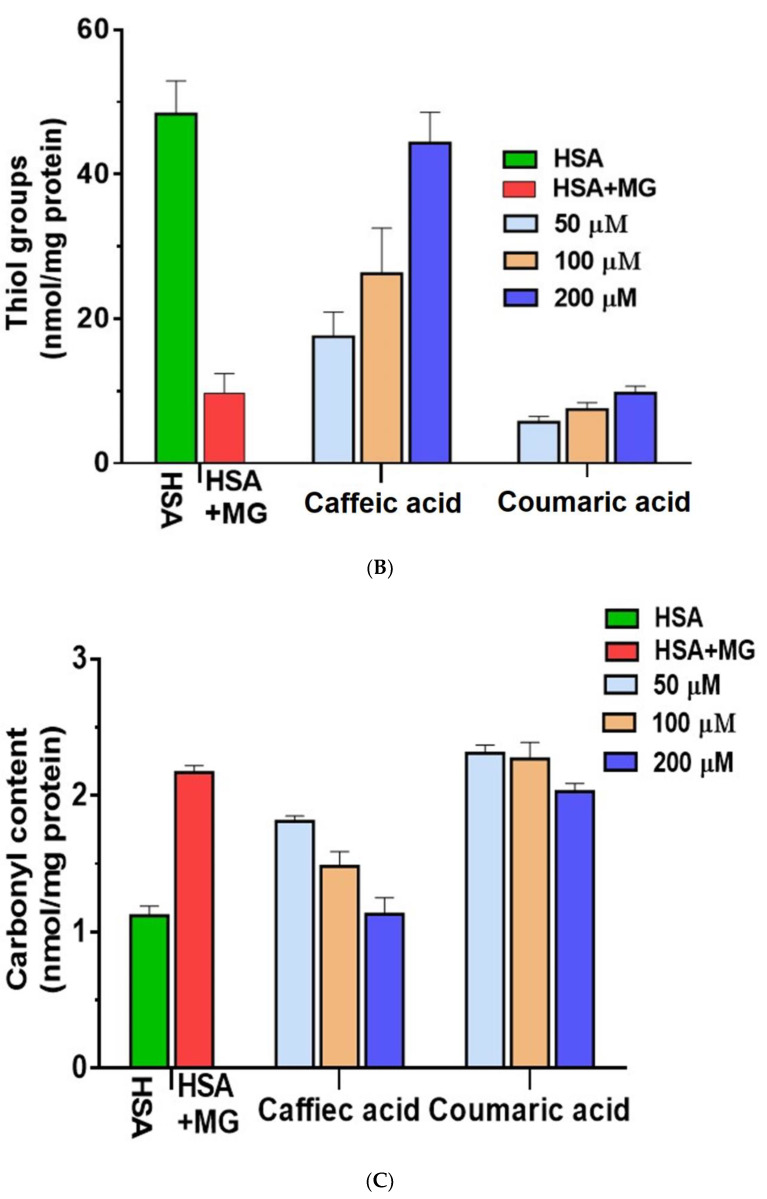
Estimation of antiglycation activity of caffeic and p-coumaric acid by monitoring (**A**) fructosamine content and protein oxidation by measuring (**B**) free thiol groups and (**C**) carbonyl content.

Fructosamine is formed by covalent attachments of sugar molecule glucose to a primary amine, followed by isomerization. The molecule undergoes Amadori rearrangement and is an indicator of early glycation products. Thus, we aimed to understand the role of caffeic acid and coumaric acid in the inhibition of glycation. Figure 5A indicates the level of fructosamine in different samples.

In control HSA, the fructosamine level was nearly 22.12 nmol/mg protein. However, incubation of HSA with MG showed a hike in fructosamine content to 114.63 nmol/mg protein (Table 3). Fructosamine levels showed a decline with successive increases in ligand concentration. In the presence of 200 μM caffeic acid, the fructosamine level declined to 46.13 nmol/mg, while coumaric acid declined the levels of fructosamine to 96.81 nmol/mg. The results showed that early end product formation viz fructosamine declines in the presence of both the ligands. Amadori product accumulations are associated with diabetic complications, and the selected natural polyphenols have the potential to reduce fructosamine content. The correlation shows the importance of caffeic acid and coumarin in managing complications due to diabetes.

### 3.5. Free–SH Groups Content

Free sulfhydryl group alterations are an important parameter to estimate oxidative modification. A disulfide bond between the sulfhydryl groups is formed due to oxidation. The oxidative modifications in HSA resultant of glycation were correlated with that of free sulfhydryl content. Figure 5B shows the free thiol group estimated for native HSA, protein incubated with MG and different concentrations of caffeic acid and coumarin. Native HSA was used as a control and was found to have 48.23 nmol/mg protein, while MG treated protein had 6.68 nmol/mg protein. Free sulfhydryl contents relate directly to the oxidation of HSA [53]. With the increase in the concentration of both the ligands, an increase in the free thiol group of HSA was observed. The maximum value was obtained at 200 μM of caffeic acid and coumaric acid. Caffeic acid increased the content of thiol to 17.8, 26.2 and 44.3 nmol/mg for 50, 100 and 200 μM, respectively. Similarly, for the same concentration of coumaric acid, the values were 5.8, 7.2 and 9.7 nmol/mg, respectively (Table 3 and Table 4).

Glycation reactions generate ROS that works against the oxidative defense mechanism of the protein group [57]. Thus, the above observations show that caffeic acid and coumarin have the potential to reduce free thiol group, resisting glycation. The results that are presented here are supported by previously published results [29,57,58].

### 3.6. Carbonyl Content

Glycation is a non-enzymatic reaction involving protein and sugar. It results in the formation of an unstable Schiff base, further leading to ketoamine production [33]. HSA was incubated with MG and was studied for its carbonyl content. Native HSA and MG-incubated HSA showed differences in their concentration by more than double. Native HSA was estimated 1.12 nmol/mg, while glycated HSA had 2.17 nmol/mg carbonyl content. With successive addition of caffeic acid and coumaric acid, carbonyl content decreased slightly (Figure 5C, Table 3 and Table 4).

### 3.7. Molecular Docking and Dynamics

**Figure 6:**
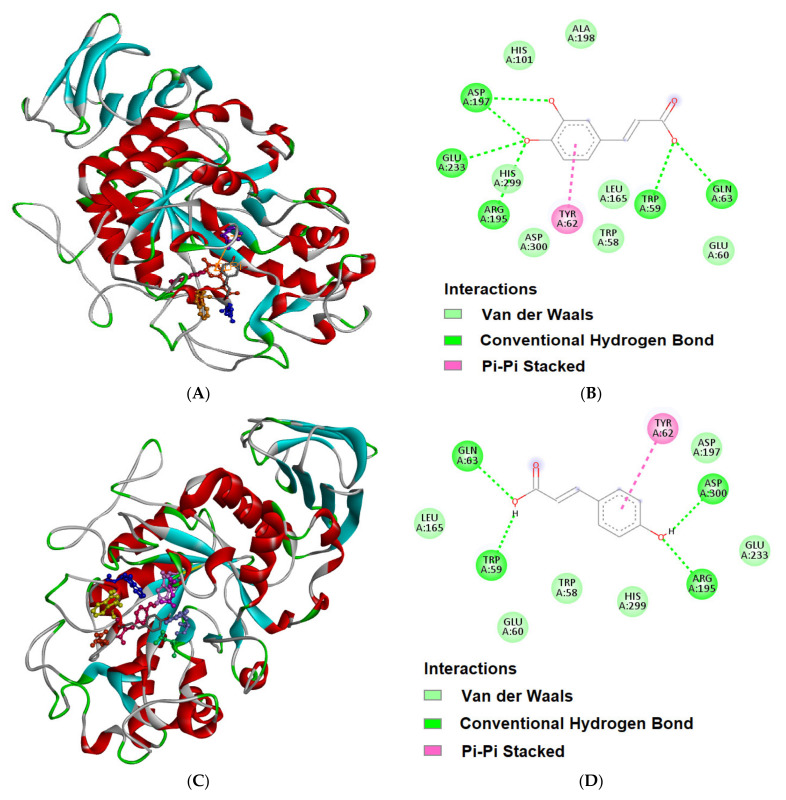
Molecular docking of caffeic acid with pancreatic α-amylase. (**A**) Binding of caffeic acid at the catalytic site of pancreatic α-amylase. (**B**) Amino acid residues and the types of forces in stabilizing the pancreatic α-amylase–caffeic acid complex (Discovery Studio). Similarly, for p-coumaric acid with α-amylase (Panels (**C**,**D**)).

The interaction between pancreatic α-amylase and both caffeic acid and p-coumaric acid was performed using Autodock 4.2.6. Molecular docking has been widely utilized to study the critical residues and sites involved in protein–ligand interactions. Our in vitro results demonstrated the mode of binding between α-amylase and polyphenols (caffeic and coumaric acid), and these are further validated by employing docking studies. Figure 6A depicts the three-dimensional structure of α-amylase in cartoon form with caffeic acid shown in the catalytic pocket depicted in balls and stick model. Caffeic acid formed six hydrogen bonds (Trp 59, Gln 63, Arg 195, Arg 195, Asp 197 and Asp 197) and three hydrophobic interactions (Trp 58, Trp 59 and Tyr 62) with α-amylase (Figure 6B, Table 5) showing a binding affinity of −5.09 kcal/mol. In comparison, coumaric acid formed H-bonds with Trp 59, Gln, 63, Arg 195, Aand sp 300 (Table 6) and shared the common hydrophobic residues as of caffeic acid.

The binding affinity observed for the interaction of coumaric acid to α-amylase was −5.04 kcal/mol. It is apparent that caffeic acid forms six hydrogen bonds as compared to three hydrogen bonds for coumaric acid, revealing that the binding of caffeic acid binds to α-amylase is more significant, validating our earlier observations.

## 4. Conclusions

**Figure 7:**
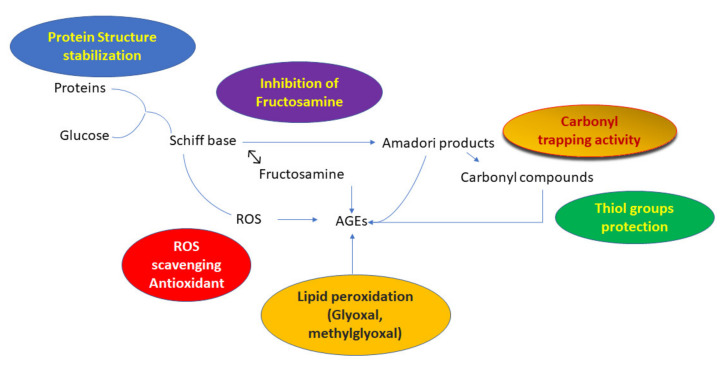
Mechasnidtic pathway of caffeic and coumaric acid to inhibit advanced glycation end-products (AGEs).

Enzymes such as amylase break down polysaccharides into monomeric sugars and thereby increase glucose concentrations in the serum. Furthermore, prolonged exposure of proteins to sugars and dicarbonyl intermediates led to the formation of advanced glycation end-products (AGEs). In our study, neutraceuticals molecules such as caffeic and coumaric acid bind with α-amylase and also inhibit the AGEs formation to a different extent. Caffeic acid possesses more inhibitory activity, which could be due to its planarity and hydrogen bonding potential. Van der Waals and hydrogen bonding are the major forces between the polyphenols–protein interactions. Molecular docking along with fluorescence quenching and synchronous fluorescence displayed the ability of phenolics to form stable complex with amylase. Moreover, these phenolics decrease AGE formation by inhibiting fructosamine. Furthermore, oxidation of proteins boosted the effect of glycation; caffeic and coumaric acid attenuate it by protecting thiol and carbonyl groups. The scheme for our probable mechanism for AGE inhibition is depicted in Figure 7. More research on similar structures along with in vivo studies is warranted to design inhibitors for diabetic complications.

## Figures and Tables

**Table 1:** Binding and thermodynamic parameters obtained for caffeic acid–α- amylase interaction obtained through fluorescence spectroscopy.

Temp. (°C)	*K*_sv_ (10^4^ M^−1^)	*K*_q_ (10^13^ M^−1^ s^−1^)	*K* (10^4^ M^−1^)	Δ*G* (kcal mol^−1^)	Δ*S* (cal.mol^−1^K^−1^)	Δ*H* (kcal mol^−1^)	TΔS (kcal mol^−1^)
25	2.1	0.77	5.37	−6.49	−16.04	−11.28	−4.78
30	1.4	0.51	5.12	−6.41			−4.86
35	0.9	0.33	2.88	−6.33			−4.94

**Table 2:** Binding and thermodynamic parameters obtained for coumaric acid–α- amylase interaction obtained through fluorescence spectroscopy.

Temp. (°C)	*K*_sv_ (10^4^ M^−1^)	*K*_q_ (10^13^ M^−1^ s^−1^)	*K* (10^4^ M^−1^)	Δ*G* (kcal mol^−1^)	Δ*S* (cal.mol^−1^K^−1^)	Δ*H* (kcal mol^−1^)	TΔS (kcal mol^−1^)
25	1.6	0.59	0.2	−4.65	178.11	48.42	53.07
30	1.3	0.48	1.5	−5.54			53.96
35	1.1	0.40	2.9	−6.43			54.85

**Table 3:** Effect of different concentrations of caffeic acid on alpha amylase.

Group	(nmol/mg Protein)		
	Fructosamine	Thiol Groups (SH)	Carbonyl Content
Control	22.12 ± 2.3	48.23 ± 7.8	1.12 ± 0.06
Glycated	114.63 ± 6.8	6.86 ± 0.64	2.17 ± 0.08
Caffeic acid:			
50 µM	82.41 ± 7.2	17.74 ± 3.4	1.81 ± 0.07
100 µM	67.34 ± 6.3	26.24 ± 1.8	1.48 ± 0.11
200 µM	46.63 ± 5.6	44.31 ± 4.7	1.13 ± 0.07

**Table 4:** Effect of different concentrations of p-coumaric acid on alpha amylase.

Group	(nmol/mg Protein)		
	Fructosamine	Thiol Groups (SH)	Carbonyl Content
Control	22.12 ± 2.3	48.23 ± 7.8	1.12 ± 0.06
Glycated	114.63 ± 6.8	6.86 ± 0.64	2.17 ± 0.08
p-coumaric acid:			
50 µM	106.68 ± 6.4	5.82 ± 0.54	2.31 ± 0.03
100 µM	103.42 ± 7.3	7.16 ± 0.41	2.27 ± 0.12
200 µM	96.81 ± 6.6	9.72 ± 0.63	2.03 ± 0.06

**Table 5:** Molecular docking parameters for caffeic acid–pancreatic α- amylase interactions obtained through (PLIP).

Hydrophobic Interactions		Hydrogen Bonds		Pi-Stacking			Docking Energy (kcal mol^−1^)
AA	Distance (Å)	AA	Distance (Å)	AA	Distance (Å)	Type	−5.09
TRP58	3.18	TRP59	1.83	TYR62	3.91	Parallel	
TRP59	3.67	GLN63	1.86				
TYR62	3.35	ARG195	2.85				
		ARG195	2.29				
		ASP197	1.85				

**Table 6:** Molecular docking parameters for p-coumaric acid–pancreatic α- amylase interactions obtained through PLIP.

Hydrophobic. Interactions		Hydrogen Bonds		Pi-Stacking			Docking Energy (kcal mol^−1^)
AA	Distance (Å)	AA	Distance (Å)	AA	Distance (Å)	Type	−5.04
TRP58	3.64	TRP59	2.65	TYR62	4.14	Parallel	
TRP59	3.54	GLN63	3.14				
TYR62	3.33	ARG195	3.69				
		ARG195	2.99				
		ASP197	3.15				
		HIS299	3.66				
		ASP300	2.91				

## Data Availability

Data will be available on request to corresponding author.

## References

[1] Sapra A., Bhandari P. (2019). Diabetes Mellitus.

[2] Sabharwal R., Mahajan A. (2020). Diabetes mellitus, dyslipidemia: Cause for acute myocardial infarction. JK Sci..

[3] Dubey S., Ganeshpurkar A., Ganeshpurkar A., Bansal D., Dubey N. (2017). Glycolytic enzyme inhibitory and antiglycation potential of rutin. Future J. Pharm. Sci..

[4] Younus H., Anwar S. (2016). Prevention of non-enzymatic glycosylation (glycation): Implication in the treatment of diabetic complication. Int. J. Health Sci..

[5] Zhang Q., Ames J.M., Smith R.D., Baynes J.W., Metz T.O. (2009). A perspective on the Maillard reaction and the analysis of protein glycation by mass spectrometry: Probing the pathogenesis of chronic disease. J. Proteome Res..

[6] Neglia C.I., Cohen H.J., Garber A.R., Ellis P.D., Thorpe S.R., Baynes J.W. (1983). 13C NMR investigation of nonenzymatic glucosylation of protein. Model studies using RNase A. J. Biol. Chem..

[7] Baynes J., Watkins N., Fisher C., Hull C., Patrick J., Ahmed M., Dunn J., Thorpe S. (1989). The Amadori product on protein: Structure and reactions. Prog. Clin. Biol. Res..

[8] Han D., Yamamoto Y., Munesue S., Motoyoshi S., Saito H., Win M.T.T., Watanabe T., Tsuneyama K., Yamamoto H. (2013). Induction of receptor for advanced glycation end products by insufficient leptin action triggers pancreatic β-cell failure in type 2 diabetes. Genes Cells.

[9] Di Meo S., Reed T.T., Venditti P., Victor V.M. (2018). Harmful and beneficial role of ROS 2017. Oxidative Med. Cell. Longev..

[10] Reddy V.P., Beyaz A. (2006). Inhibitors of the Maillard reaction and AGE breakers as therapeutics for multiple diseases. Drug Discov. Today.

[11] Kalousova M., Skrha J., Zima T. (2002). Advanced glycation end-products and advanced oxidation protein products in patients with diabetes mellitus. Physiol. Res..

[12] Fu M.-X., Requena J.R., Jenkins A.J., Lyons T.J., Baynes J.W., Thorpe S.R. (1996). The Advanced Glycation End Product, N∊-(Carboxymethyl) lysine, Is a Product of both Lipid Peroxidation and Glycoxidation Reactions (∗). J. Biol. Chem..

[13] Willemsen S., Hartog J.W., Heiner-Fokkema M.R., van Veldhuisen D.J., Voors A.A. (2012). Advanced glycation end-products, a pathophysiological pathway in the cardiorenal syndrome. Heart Fail. Rev..

[14] Singh V.P., Bali A., Singh N., Jaggi A.S. (2014). Advanced glycation end products and diabetic complications. Korean J. Physiol. Pharmacol..

[15] Brownlee M. (1995). The pathological implications of protein glycation, Clinical and investigative medicine. Med. Clin. Exp..

[16] Kotowaroo M., Mahomoodally M., Gurib-Fakim A., Subratty A. (2006). Screening of traditional antidiabetic medicinal plants of mauritius for possible α-amylase inhibitory effects in vitro. Phytother. Res. Int. J. Devoted Pharmacol. Toxicol. Eval. Nat. Prod. Deriv..

[17] Kim Y.-M., Jeong Y.-K., Wang M.-H., Lee W.-Y., Rhee H.-I. (2005). Inhibitory effect of pine extract on α-glucosidase activity and postprandial hyperglycemia. Nutrition.

[18] Shamsi A., Mohammad T., Anwar S., Alajmi M.F., Hussain A., Hassan M.I., Ahmad F., Islam A. (2020). Probing the interaction of Rivastigmine Tartrate, an important Alzheimer’s drug, with serum albumin: Attempting treatment of Alzheimer’s disease. Int. J. Biol. Macromol..

[19] Anwar S., Shamsi A., Mohammad T., Islam A., Hassan M.I. (2021). Targeting pyruvate dehydrogenase kinase signaling in the development of effective cancer therapy. Biochim. Biophys. Acta Rev. Cancer.

[20] Anwar S., Mohammad T., Shamsi A., Queen A., Parveen S., Luqman S., Hasan G.M., Alamry K.A., Azum N., Asiri A.M. (2020). Discovery of Hordenine as a potential inhibitor of pyruvate dehydrogenase kinase 3: Implication in lung Cancer therapy. Biomedicines.

[21] Anwar S., Khan S., Anjum F., Shamsi A., Khan P., Fatima H., Shafie A., Islam A., Hassan M.I. (2021). Myricetin inhibits breast and lung cancer cells proliferation via inhibiting MARK4. J. Cell. Biochem..

[22] Anwar S., Shamsi A., Kar R.K., Queen A., Islam A., Ahmad F., Hassan M.I. (2020). Structural and biochemical investigation of MARK4 inhibitory potential of cholic acid: Towards therapeutic implications in neurodegenerative diseases. Int. J. Biol. Macromol..

[23] Anwar S., Khan S., Shamsi A., Anjum F., Shafie A., Islam A., Ahmad F., Hassan M.I. (2021). Structure-based investigation of MARK4 inhibitory potential of Naringenin for therapeutic management of cancer and neurodegenerative diseases. J. Cell. Biochem..

[24] Khandouzi N., Zahedmehr A., Nasrollahzadeh J. (2021). Effect of polyphenol-rich extra-virgin olive oil on lipid profile and inflammatory biomarkers in patients undergoing coronary angiography: A randomised, controlled, clinical trial. Int. J. Food Sci. Nutr..

[25] Rienks J., Barbaresko J., Oluwagbemigun K., Schmid M., Nöthlings U. (2018). Polyphenol exposure and risk of type 2 diabetes: Dose-response meta-analyses and systematic review of prospective cohort studies. Am. J. Clin. Nutr..

[26] Martin K.R., Appel C.L. (2009). Polyphenols as dietary supplements: A double-edged sword. Nutr. Diet. Suppl..

[27] Magnani C., Isaac V.L.B., Correa M.A., Salgado H.R.N. (2014). Caffeic acid: A review of its potential use in medications and cosmetics. Anal. Methods.

[28] Grabska-Kobylecka I., Kaczmarek-Bak J., Figlus M., Prymont-Przyminska A., Zwolinska A., Sarniak A., Wlodarczyk A., Glabinski A., Nowak D. (2020). The presence of caffeic acid in cerebrospinal fluid: Evidence that dietary polyphenols can cross the blood-brain barrier in humans. Nutrients.

[29] Kong C.S., Jeong C.H., Choi J.S., Kim K.J., Jeong J.W. (2013). Antiangiogenic effects of p-coumaric acid in human endothelial cells. Phytother. Res..

[30] El-Seedi H.R., El-Said A.M., Khalifa S.A., Goransson U., Bohlin L., Borg-Karlson A.-K., Verpoorte R. (2012). Biosynthesis, natural sources, dietary intake, pharmacokinetic properties, and biological activities of hydroxycinnamic acids. J. Agric. Food Chem..

[31] Pei K., Ou J., Huang J., Ou S. (2016). p-Coumaric acid and its conjugates: Dietary sources, pharmacokinetic properties and biological activities. J. Sci. Food Agric..

[32] Shamsi A., Mohammad T., Khan M.S., Shahwan M., Husain F.M., Rehman M., Hassan M., Ahmad F., Islam A. (2019). Unraveling binding mechanism of Alzheimer’s drug rivastigmine tartrate with human transferrin: Molecular docking and multi-spectroscopic approach towards neurodegenerative diseases. Biomolecules.

[33] Shamsi A., Ahmed A., Khan M.S., Husain F.M., Bano B. (2020). Rosmarinic acid restrains protein glycation and aggregation in human serum albumin: Multi spectroscopic and microscopic insight-possible therapeutics targeting diseases. Int. J. Biol. Macromol..

[34] Khan M.S., Rehman M.T., Ismael M.A., AlAjmi M.F., Alruwaished G.I., Alokail M.S., Khan M.R. (2021). Bioflavonoid (Hesperidin) Restrains Protein Oxidation and Advanced Glycation End Product Formation by Targeting AGEs and Glycolytic Enzymes. Cell Biochem. Biophys..

[35] Shamsi A., Ahmed A., Khan M.S., al Shahwan M., Husain F.M., Bano B. (2020). Understanding the binding between Rosmarinic acid and serum albumin: In vitro and in silico insight. J. Mol. Liq..

[36] Ahmed A., Shamsi A., Khan M.S., Husain F.M., Bano B. (2018). Methylglyoxal induced glycation and aggregation of human serum albumin: Biochemical and biophysical approach. Int. J. Biol. Macromol..

[37] Shamsi A., Anwar S., Shahbaaz M., Mohammad T., Alajmi M.F., Hussain A., Hassan I., Ahmad F., Islam A. (2020). Evaluation of Binding of Rosmarinic Acid with Human Transferrin and Its Impact on the Protein Structure: Targeting Polyphenolic Acid-Induced Protection of Neurodegenerative Disorders. Oxidative Med. Cell. Longev..

[38] Khan S.N., Islam B., Yennamalli R., Sultan A., Subbarao N., Khan A.U. (2008). Interaction of mitoxantrone with human serum albumin: Spectroscopic and molecular modeling studies. Eur. J. Pharm. Sci..

[39] Pacheco M.E., Bruzzone L. (2013). Synchronous fluorescence spectrometry: Conformational investigation or inner filter effect?. J. Lumin..

[40] Shamsi A., Shahwan M., Husain F.M., Khan M.S. (2019). Characterization of methylglyoxal induced advanced glycation end products and aggregates of human transferrin: Biophysical and microscopic insight. Int. J. Biol. Macromol..

[41] Giannoukakis N. (2006). Drug evaluation: Ranirestat—An aldose reductase inhibitor for the potential treatment of diabetic complications. Curr. Opin. Investig. Drugs.

[42] Khan M.S., Qais F.A., Rehman M.T., Ismail M.H., Alokail M.S., Altwaijry N., Alafaleq N.O., AlAjmi M.F., Salem N., Alqhatani R. (2020). Mechanistic inhibition of non-enzymatic glycation and aldose reductase activity by naringenin: Binding, enzyme kinetics and molecular docking analysis. Int. J. Biol. Macromol..

[43] Johnson R.N., Metcalf P.A., Baker J.R. (1983). Fructosamine: A new approach to the estimation of serum glycosylprotein. An index of diabetic control. Clin. Chim. Acta.

[44] Ellman G.L. (1958). A colorimetric method for determining low concentrations of mercaptans. Arch. Biochem. Biophys..

[45] Brayer G.D., Luo Y., Withers S.G. (1995). The structure of human pancreatic α-amylase at 1.8 Å resolution and comparisons with related enzymes. Protein Sci..

[46] Zhang H., Wang Y., Fei Z., Wu L., Zhou Q. (2008). Characterization of the interaction between Fe (III)-2, 9, 16, 23-tetracarboxyphthalocyanine and blood proteins. Dye. Pigment..

[47] Wang Q., Huang C.-R., Jiang M., Zhu Y.-Y., Wang J., Chen J., Shi J.-H. (2016). Binding interaction of atorvastatin with bovine serum albumin: Spectroscopic methods and molecular docking. Spectrochim. Acta Part A Mol. Biomol. Spectrosc..

[48] Zhang Y.-F., Zhou K.-L., Lou Y.-Y., Pan D.-Q., Shi J.-H. (2017). Investigation of the binding interaction between estazolam and bovine serum albumin: Multi-spectroscopic methods and molecular docking technique. J. Biomol. Struct. Dyn..

[49] Wang B.-L., Pan D.-Q., Zhou K.-L., Lou Y.-Y., Shi J.-H. (2019). Multi-spectroscopic approaches and molecular simulation research of the intermolecular interaction between the angiotensin-converting enzyme inhibitor (ACE inhibitor) benazepril and bovine serum albumin (BSA). Spectrochim. Acta Part A Mol. Biomol. Spectrosc..

[50] Shamsi A., Mohammad T., Anwar S., Nasreen K., Hassan M.I., Ahmad F., Islam A. (2020). Insight into the binding of PEG-400 with eye protein alpha-crystallin: Multi spectroscopic and computational approach: Possible therapeutics targeting eye diseases. J. Biomol. Struct. Dyn..

[51] Ross P.D., Subramanian S. (1981). Thermodynamics of protein association reactions: Forces contributing to stability. Biochemistry.

[52] Ahmed A., Shamsi A., Khan M.S., Husain F.M., Bano B. (2019). Probing the interaction of human serum albumin with iprodione, a fungicide: Spectroscopic and molecular docking insight. J. Biomol. Struct. Dyn..

[53] Chao C.Y., Mong M.C., Chan K.C., Yin M.C. (2010). Anti-glycative and anti-inflammatory effects of caffeic acid and ellagic acid in kidney of diabetic mice. Mol. Nutr. Food Res..

[54] Moselhy S.S., Razvi S.S., Lshibili F.A.A., Kuerban A., Hasan M.N., Balamash K.S., Huwait E.A., Abdulaal W.H., Al-Ghamdi M.A., Kumosani T.A. (2018). m-Coumaric acid attenuates non-catalytic protein glycosylation in the retinas of diabetic rats. J. Pestic. Sci..

[55] Shiozawa R., Inoue Y., Murata I., Kanamoto I. (2018). Effect of antioxidant activity of caffeic acid with cyclodextrins using ground mixture method. Asian J. Pharm. Sci..

[56] Liu J., He Y., Wang S., He Y., Wang W., Li Q., Cao X. (2018). Ferulic acid inhibits advanced glycation end products (AGEs) formation and mitigates the AGEsinduced inflammatory response in HUVEC cells. J. Funct. Foods.

[57] Wu C.-H., Huang H.-W., Lin J.-A., Huang S.-M., Yen G.C. (2011). The proglycation effect of caffeic acid leads to the elevation of oxidative stress and inflammation in monocytes, macrophages and vascular endothelial cells. J. Nutr. Biochem..

[58] Ying X., Meng Z., Weiyu W., Yin H., Jianli L. (2019). Caffeic Acid Inhibits the Formation of Advanced Glycation End Products (AGEs) and Mitigates the AGEs-Induced Oxidative Stress and Inflammation Reaction in Human Umbilical Vein Endothelial Cells (HUVECs). Chem. Biodivers..

